# Generative AI in drug discovery and development: the next revolution of drug discovery and development would be directed by generative AI

**DOI:** 10.1097/MS9.0000000000002438

**Published:** 2024-08-14

**Authors:** Chiranjib Chakraborty, Manojit Bhattacharya, Soumen Pal, Md. Aminul Islam

**Affiliations:** a Department of Biotechnology, School of Life Science and Biotechnology, Adamas University, Kolkata, West Bengal; b Department of Zoology, Fakir Mohan University, Vyasa Vihar, Balasore, Odisha; c School of Mechanical Engineering, Vellore Institute of Technology, Vellore, Tamil Nadu, India; d COVID-19 Diagnostic Lab, Department of Microbiology, Noakhali Science and Technology University, Noakhali; e Advanced Molecular Lab, Department of Microbiology, President Abdul Hamid Medical College, Karimganj, Kishoreganj, Bangladesh

*Dear Editor,*

Previously, we published a correspondence article in International Journal of Surgery about next-generation drug discovery and development using ChatGPT or Large Language Model (LLM)^1^. The article was very timely. However, we found that generative artificial intelligence (AI) is progressing quickly in drug discovery and development. In this article, we discuss the role of generative AI in drug discovery and development.

## Background: Generative AI in drug discovery and development

The drug discovery process is an expensive, time-consuming, and risk-associated process. The process requires US$2.5 billion and around 12–15 years to obtain a novel drug into the market^2,3^. The initial research usually occurs in academia, yielding data to develop a hypothesis for a new drug. Presently, drug discovery starts with drug target discovery. AI has a significant role in drug target discovery^4^. However, a large number of drug targets are protein-based. One significant protein-based drug target class is G-protein coupled receptor (GPCR). Drugs targeting the GPCR class contain angiotensin receptor blockers, β-blockers, opioid agonists, and histamine receptor blockers. Commonly used β-blockers include metoprolol, propranolol, atenolol, bisoprolol, etc. During drug development, a pathway’s protein activation or inhibition might help to understand the therapeutic effect of a molecule during drug development using a protein-based drug target. The drug-like properties of the chemical compound might be further explored during drug development^5^. A paradigm shift has been documented in the last few years in the field of drug discovery and development using computational resources and technologies. Computational technologies generate vast amounts of data on drug-like properties of the chemical compound, which can bind to therapeutic targets. At the same time, computational technologies help to develop 3D structures of the therapeutic targets. Many computing capacities to help generate a vast amount of data. Fast computational methodologies have been developed using computational resources and technologies. One example is structure-based virtual screening using Giga scale chemical spaces. In this direction, some technologies have been developed for ultra-large virtual screens. Here, the technology screened millions to billions of compounds. Another example is screening ligand characteristics and analyzing drug target activities using deep learning (DL) methodologies.

Recently, AI-based technologies have been helping to speed up the drug discovery and development process. AI-derived drug molecules show a chemical space similar to previously published drugs. Recently, two receptors targeted, AI-derived drug molecules entered the clinic. These two drug molecules are target serotonin receptors. Examples of these two AI-derived drug molecules are a 5-HT1A agonist and a 5-HT2A antagonist, which entered the clinic^6^. AI has revolutionized the determination of accurate protein structure. Therefore, the unsolved protein-based drug target structure can be solved through AI or AI-related tools or databases, such as the AlphaFold database. AlphaFold, a state-of-the-art machine learning (ML) approach developed by DeepMind, helps to predict nearly the structure of the entire human proteome. One example is the structures of two G6Pases (G6Pase-α and G6Pase-β) with the active sites developed by researchers using the AlphaFold model^7^. The AlphaFold protein structure database has expanded to include millions of proteins, covering vast categories and revolutionising protein structure prediction^8^. It helps us to understand most of the protein-based drug targets. AlphaFold uses a deep neural network algorithm to develop the structures. However, this AlphaFold tool varies and provides benefits to researchers in areas ranging from drug discovery to medicinal chemistry. It will open a new path to work with those structures of protein-based drug targets that were previously unsolved. Boston Consulting Group (BCG), Massachusetts, USA, reported that 20 relatively new AI-intensive pharmaceutical companies were founded between 2010 and 2021. BCG also reported that about 15 AI-developed drug candidates had entered different clinical trial stages^3,6^. Some examples of AI-designed drugs that entered clinical trials are: (i) REC-2282 is a small molecule pan-HDAC inhibitor. The indication of the drug is neurofibromatosis type 2. It is in a Phase 2/3 clinical trial and was developed by Recursion. (ii) BEN-8744 is a small molecule PDE10 inhibitor. The indication of the drug is slcerative colitis. It is in a Phase 1 clinical trial and was developed by BenevolentAI^9^. Therefore, it was noted that AI-based drug discovery and development saves time and cost by at least 25–50%^3^. Understanding the Quantitative structure-activity relationships (QSARs) is an empirical part of drug discovery. QSARs provide an understanding of the drug molecule structure and functional-activity relationship through statistical models. Currently, AI models are highly reliable in the case of QSAR and computational chemistry. DL models and big data enhance the processing of unstructured data, which helps with more potent QSAR model formation and provides a comprehensive interpretation of the drug discovery process^10,11^. Similarly, several online prediction platforms have been developed using AI/ML algorithms for drug molecule structure prediction, understanding the drug ability and characteristics of the molecule that help drug discovery and development. One example of a tool is PPICurator, an AI / ML-based tool for comprehensive data mining protein-protein interaction assessment^12^. Another example is DGIdb, an online platform for analyzing the drug-gene interaction^13^. Besides, AI technology helps in several subfields of preclinical studies, an essential aspect of drug discovery. Recent ML algorithms also benefit ADME predictions/ PBPK modeling. Therefore, AI-based computing has helped to generate different data-driven and faster tools and databases for drug discovery and development. So, AI-driven computational tools help speed up drug molecule discovery. AI-driven ChatGPT, or LLM, assists as a computational drug discovery and development tool that helps in a faster drug discovery and development process.

## Definition and explanation of generative AI

There has been an AI boom over the past few years. People are using ML models to process a vast amount of data. However, once ChatGPT was introduced, people started using ChatGPT or LLM. ChatGPT or LLM is a generative AI that can generate more objects after training the input datasets. Once trained, generative AI can generate high-quality text, images, video, music, and other content. Generative AI is a DL model. During training, the models learn the patterns of the training data.

Generative AI tools have indeed existed for some decades. However, tremendous interest has recently been generated in utilizing these tools with the success of chatbots, especially with the origin of ChatGPT. Currently, ChatGPT is used for drug discovery and development, from target to small molecule discovery^4^. However, several generative AI models have successfully performed in small-scale laboratory settings, and some researchers have questioned their performance in real-world conditions. Nevertheless, we must recognize the capability of generative AI technologies in various application areas including the drug discovery process. DL-based methods are used in all stages of drug development. Deep neural networks (DNN) and deep learning networks (DLN) related methods, part of generative AI, are currently applied in drug-related data growth. Generative AI can develop superior text, concept art, visual art, code, animation, speech, music, and video. For instance, it is possible to synthesize good-quality visual representation using diffusion models^14^. The generative power of these tools can modify the traditional creative process methodologies that developers employ to design and produce. To comprehend the impact of generative AI across numerous domains and initiate regulatory policies, stakeholders must focus on novel interdisciplinary approaches and investigate the interaction between creativity and technology^15^.

**Figure 1:**
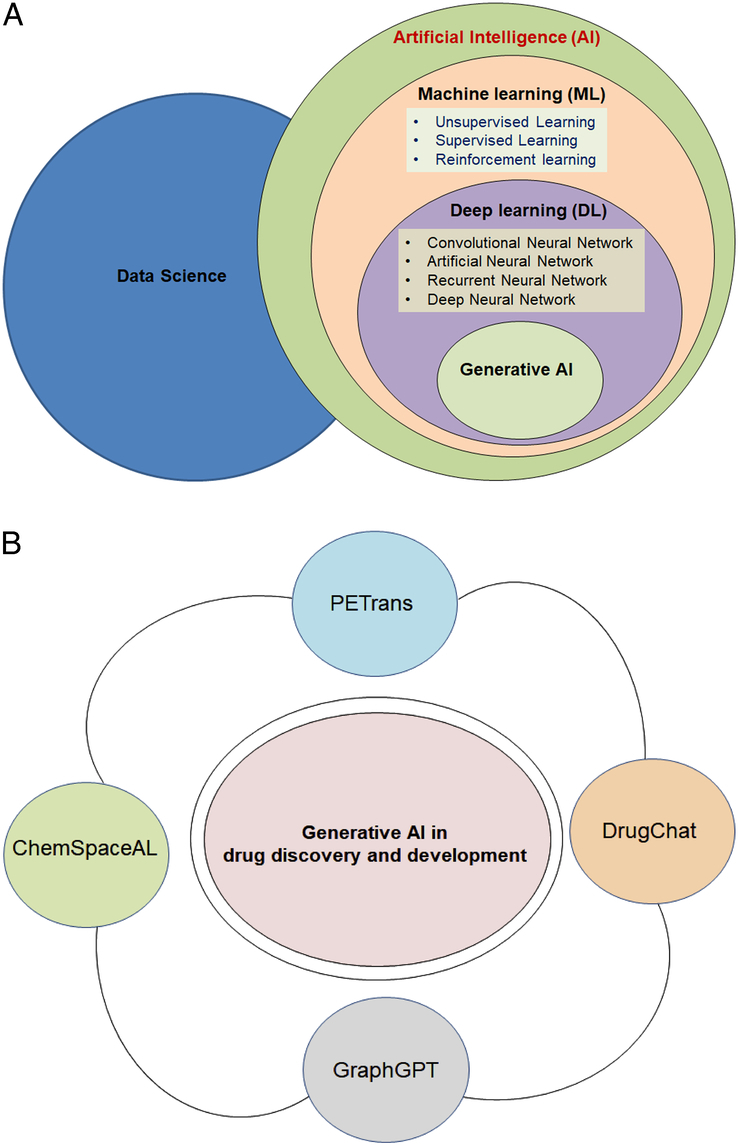
The figure depicts the schematic diagram to understand the relationship between artificial intelligence (AI) and Generative AI. The figure also depicts the application of generative AI in drug discovery and development. (A) The figure depicts the schematic diagram to show the relationship between AI, machine learning (ML), deep learning (DL), and generative AI. It also shows how these components are linked with the data science. (B) The figure depicts the application of generative AI in drug discovery and development, such as ChemSpaceAL, PETrans, DrugChat, GraphGPT, etc.

DL concept is employed for generative AI models, and DNNs constitute the most crucial part (Fig. 1A). The DNNs train on considerable datasets to understand their patterns and statistical distributions and DNNs use generative models to produce novel datasets. Scientists are also trying to develop novel datasets for drug discovery and development using DNNs. With the help of these networks, generative AI models recognize the patterns in the existing datasets to develop novel and genuine content^16^. As an advancement in generative AI, the GPT (generative pre-trained transformer) technology, a LLM, was introduced in 2018 when OpenAI launched the first version i.e GPT-1 in USA. The GPTs utilize transformer architecture and belong to a class of neural network models, and these transformers enable applications to generate human-like output and provide replies in conversational mode. GPT models have immense use in producing genuine text and other content and can be suitably employed in chatbots. The exciting advantage of GPT models is their processing speed, where the result is obtained for complex input queries in just a few seconds. New versions of the GPT model have been developed. Brynjolfsson and colleagues reported in their study that for GPT-3, the model uses ~0.3 trillion tokens and 0.175 trillion parameters for training. Moreover, for training the recent version GPT-4, the approximate number of tokens and parameters used are 13 trillion and 1.8 trillion, respectively^17^. GPT-4, a multimodal LLM, can be trained on numerous parameters compared to GPT-3. Therefore, multimodal LLM can efficiently perform drug discovery processes, from drug target discovery to small molecule design. It is expected that, this evolution of LLM to multimodal LLM will change the drug discovery landscape shortly.

## Application of generative AI in drug discovery and development

ChatGPT’s popularity and widespread use with reasonable success in a very short period has given rise to a ‘debatable storm’ on the use of generative AI in healthcare and pharmaceutical industries^4,18^. Currently, ChatGPT has over 180.5 million users. Therefore, generative -AI, or LLM, has more than 180.5 million users^19^. Generative AI models are helping to solve the critical problems of chemistry. Researchers have recently used GPT models to make International Union of Pure and Applied Chemistry (IUPAC) nomenclature or even common names as inputs during the molecular estimation job. Similarly, Kyro and colleagues developed ChemSpaceAL, the generation of protein-specific molecules. These models will help to develop a molecular generation by a GPT-based molecular generator^20^. At the same time, Lu and colleagues developed GraphGPT, which will help in condition-based molecular generation. The model will assist in developing a vast number of molecules with particular properties that can be used as a virtual drug screening library. This model will accelerate the drug discovery process and help to solve the critical problems in drug discovery^21^. Likewise, Wang and colleagues developed PETrans, which is used to design and develop novel drug molecules using deep generative models. Here, GPT was applied to extract the appropriate features of the molecule^22^. Drug-drug interactions (DDIs) are a critical area in drug discovery and development. Juhi and colleagues explored the capability of the GPT model to understand the DDIs. Using the GPT model with two-stage questions, scientists assessed 40 pairs of previously listed DDIs. They concluded that the GPT model might be an effective platform for exploring, forecasting, and explaining DDIs^23^. Recently, Liang and colleagues created DrugChat, a GPT-based pharmaceutical model, to furnish information on drug molecule graphs. The model has ChatGPT-like abilities to accelerate drug discovery, increase our understanding of aiding drug repurposing, guiding lead optimization, structure-activity relationships, streamlining clinical trials, and reducing the failure rate^24^. Therefore, GPT-based generative AI models are solving several problems of drug discovery and development (Fig. 1B). However, White explains how GPT-4 will solve more complicated problems in the different areas of chemistry, including pharmaceutical chemistry^25^. In addition to drug discovery and development, generative AI has been used in other medical and infectious disease fields, such as veterinary anatomy education^26^, travel medicine^27^, etc.

### Limitations

AI-enabled models depend on the training dataset. Generative AI-based drug discovery models are also AI-enabled and depend on the training datasets. Therefore, the accuracy of generative AI-based drug discovery models depends on the training dataset. Similarly, generative AI-based drug models and AI-enabled drug discovery and development models must undergo validation and testing to confirm their accuracy and reliability.

## Conclusion

Generative AI has been used successfully in drug discovery. Generative AI models have some limitations or shortcomings. One example is the reproducibility crisis. The reproducibility crisis also impacts target identification^28^. Another example is AlphaFold. It can only predict a single state of a protein, even when the data includes indications of multiple states and dynamic behaviors. Additionally, the accuracy of AI in capturing protein states is not always precise. Another example of generative AI is LLM. Sometimes, LLM produces misleading information and errors^29^. However, there is a lot of scope for implementing generative AI models in pharmaceutical science. Shortly, AI-based DL-associated generative AI tools will incorporate all the data and information systematically and achieve a new level of generative AI in drug discovery. Still, academia and industry must work together to make the best use of generative AI for the next revolution of drug discovery.

## Ethical approval

This article does not require any human/animal subjects to acquire such approval.

## Source of funding

This study received no specific grant from any funding agency in the public, commercial, or not-for-profit sectors.

## Author contribution

C.C.: conceptualization, data curation, investigation, writing—original draft, writing—review and editing. M.B.: validation. S.P.: validation. M.A.I.: validation. All authors critically reviewed and approved the final version of the manuscript.

## Conflicts of interest disclosure

All authors report no conflicts of interest relevant to this article.

## Research registration unique identifying number (UIN)

1. Name of the registry: Not applicable.
2. Unique Identifying number or registration ID: Not applicable.
3. Hyperlink to your specific registration (must be publicly accessible and will be checked): Not applicable.

## Guarantor

Md. Aminul Islam.

## Data availability statement

The data in this correspondence article are not sensitive in nature and is accessible in the public domain. The data are therefore available and not of a confidential nature.

## Provenance and peer review

Not commissioned, internally peer-reviewed.

## References

[1] Pal S Bhattacharya M Islam MA. ChatGPT or LLM in next-generation drug discovery and development: Pharmaceutical and biotechnology companies can make use of the artificial intelligence (AI)-based device for a faster way of drug discovery and development. Int J Surg 2023;109:4382–4384. 37707542 10.1097/JS9.0000000000000719 PMC10720782

[2] Paul SM Mytelka DS Dunwiddie CT. How to improve R&D productivity: the pharmaceutical industry’s grand challenge. Nat Rev Drug Discov 2010;9:203–214. 20168317 10.1038/nrd3078

[3] Editorial. AI’s potential to accelerate drug discovery needs a reality check. Nature 2023;622:217. 37817040 10.1038/d41586-023-03172-6

[4] Chakraborty C Bhattacharya M Lee SS. Artificial intelligence enabled ChatGPT and large language models in drug target discovery, drug discovery, and development. Mol Ther Nucleic Acids 2023;33:866–868. 37680991 10.1016/j.omtn.2023.08.009 PMC10481150

[5] Mohs RC Greig NH. Drug discovery and development: role of basic biological research. Alzheimers Dement (N Y) 2017;3:651–657. 29255791 10.1016/j.trci.2017.10.005 PMC5725284

[6] Jayatunga MKP Xie W Ruder L. AI in small-molecule drug discovery: a coming wave? Nat Rev Drug Discov 2022;21:175–176. 35132242 10.1038/d41573-022-00025-1

[7] Tunyasuvunakool AdlerJ K Wu Z. Highly accurate protein structure prediction for the human proteome. Nature 2021;596:590–596. 34293799 10.1038/s41586-021-03828-1 PMC8387240

[8] Mullard A. What does AlphaFold mean for drug discovery? Nat Rev Drug Discov 2021;20:725–727. 34522032 10.1038/d41573-021-00161-0

[9] Arnold C. Inside the nascent industry of AI-designed drugs. Nat Med 2023;29:1292–1295. 37264208 10.1038/s41591-023-02361-0

[10] Mao J Akhtar J Zhang X. Comprehensive strategies of machine-learning-based quantitative structure-activity relationship models. iScience 2021;24:103052. 34553136 10.1016/j.isci.2021.103052 PMC8441174

[11] Tripathi MK Nath A Singh TP. Evolving scenario of big data and Artificial Intelligence (AI) in drug discovery. Mol Divers 2021;25:1439–1460. 34159484 10.1007/s11030-021-10256-w PMC8219515

[12] Li M He Q Ma J. PPICurator: a tool for extracting comprehensive protein-protein interaction information. Proteomics 2019;19:e1800291. 30521143 10.1002/pmic.201800291

[13] Cannon M Stevenson J Stahl K. DGIdb 5.0: rebuilding the drug-gene interaction database for precision medicine and drug discovery platforms. Nucleic Acids Res 2024;52:D1227–D1235. 37953380 10.1093/nar/gkad1040 PMC10767982

[14] Rombach R Blattmann A Lorenz D. High-resolution image synthesis with latent diffusion models. in Proceedings of the IEEE/CVF conference on computer vision and pattern recognition. 2022:10684–10695. 10.48550/arXiv.2112.10752

[15] Epstein Z Hertzmann A. Art and the science of generative AI. Science 2023;380:1110–1111. 37319193 10.1126/science.adh4451

[16] Lv Z. Generative Artificial Intelligence in the Metaverse Era. Cognitive Robotics; 2023.

[17] Brynjolfsson E Li D Raymond LR. Generative AI at work. National Bureau of Economic Research; 2023. 24. doi:10.3386/w31161

[18] Chakraborty C Pal S Bhattacharya M. Overview of Chatbots with special emphasis on artificial intelligence-enabled ChatGPT in medical science. Front Artif Intell 2023;6:1237704. 38028668 10.3389/frai.2023.1237704 PMC10644239

[19] Payne DL Purohit K Borrero WM. Performance of GPT-4 on the American College of Radiology In-training Examination: Evaluating Accuracy, Model Drift, and Fine-tuning. Acad Radiol 2024;31:3046–3054. 38653599 10.1016/j.acra.2024.04.006

[20] Kyro GW Morgunov A Brent RI. ChemSpaceAL: an efficient active learning methodology applied to protein-specific molecular generation. ArXiv 2023;123:283a. 10.1021/acs.jcim.3c01456 38287889

[21] Lu H Wei Z Wang X. GraphGPT: a graph enhanced generative pretrained transformer for conditioned molecular generation. Int J Mol Sci 2023;24:16761. 38069085 10.3390/ijms242316761 PMC10706000

[22] Wang X Gao C Han P. PETrans: de novo drug design with protein-specific encoding based on transfer learning. Int J Mol Sci 2023;24:1146. 36674658 10.3390/ijms24021146 PMC9865828

[23] Juhi A Pipil N Santra S. The capability of ChatGPT in predicting and explaining common drug-drug interactions. Cureus 2023;15:e36272. 37073184 10.7759/cureus.36272 PMC10105894

[24] Liang Y Zhang R Zhang L. DrugChat: towards enabling ChatGPT-like capabilities on drug molecule graphs. arXiv preprint arXiv 2023;2309:03907.

[25] White AD. The future of chemistry is language. Nat Rev Chem 2023;7:457–458. 37208543 10.1038/s41570-023-00502-0

[26] Choudhary OP Saini J Challana A. ChatGPT for veterinary anatomy education: an overview of the prospects and drawbacks. Int J Morphol 2023;41:1198–1202.

[27] Choudhary OP. ChatGPT in travel medicine: a friend or foe? Travel Med Infect Dis 2023;24:102615. 10.1016/j.tmaid.2023.102615 37399881

[28] Hasselgren C Oprea TI. Artificial intelligence for drug discovery: are we there yet? Annu Rev Pharmacol Toxicol 2023;64:527–550. 37738505 10.1146/annurev-pharmtox-040323-040828

[29] Jo A. The promise and peril of generative AI. Nature 2023;614:214–216.36747115

